# Immune System Development—Pro Tips for Paediatricians

**DOI:** 10.1111/apa.70627

**Published:** 2026-05-28

**Authors:** Petter Brodin

**Affiliations:** ^1^ Department of Women's and Children's Health Karolinska Institutet Solna Sweden; ^2^ Department of Immunology and Inflammation Imperial College London London UK; ^3^ Medical Research Council Laboratory of Medical Sciences (LMS) Imperial College Hammersmith Campus London UK

**Keywords:** allergies, asthma, immune system development, inflammation, microbiome, paediatric immunology

## Abstract

There are many misconceptions about the immune system in children, its impact on infectious disease susceptibility and its role in the rapidly increasing rates of immune mediated diseases in industrialized societies. In this mini review, I will discuss common believes and the data supporting or countering such believes, with a focus on clinically relevant topics of relevance to trainees and practicing paediatricians. I will emphasize data from studies performed in humans rather than reductionist mouse models. I discuss open questions requiring further investigation and end with a discussion on clinical observations of particular concern which paediatricians should consider when caring for children in their practices. Immune development is increasingly recognized as an important determinant of health, not just early in life, but also long term and all of us caring for children should know more about it.

## Summary

- Human immune systems are shaped by environmental exposures early in life.
- Mechanisms of early life imprinting are unknown but subject to intense investigation.
- Immune systems of young children are not deficient but naïve and calibrated differently from adult immunity and adapted to the needs of children to establish symbiotic immune‐microbe interactions for long‐term health.

## Graphical abstract

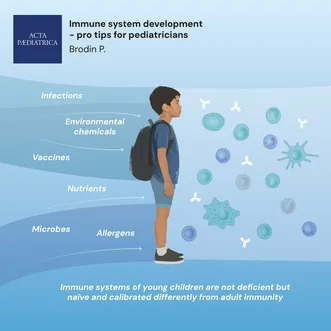

## 1 Introduction

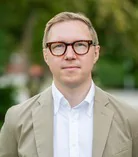

The immune system of a newborn child is immediately confronted with a vast array of environmental factors upon transitioning from the uterus which is largely sterile [1]. Within seconds of birth, the child begins breathing air containing environmental chemicals, variable amounts of pollutants and irritants associated with life in industrialized societies. The skin, the gut and airway epithelia are also colonized by microbes during the hours, days and weeks following birth. Specialized immune cells within tissues are either present already in foetal life, or infiltrate after birth and will sense each environmental factor crossing the barriers and determine whether to tolerate or reject these. Despite the recognized importance of this process, it remains insufficiently understood. We lack mechanistic understanding of what factors are most critical, what regulates variable responses to these initial encounters and how such early encounters impact immune function long‐term. This gap in our understanding is largely due to the difficulty in obtaining longitudinal samples from healthy newborn children and consequently, most of our knowledge stems from laboratory mice reared in nearly sterile environments, poorly representing the many exposures in human newborns.

The human genome evolved under strong pressures from ancestral microbes, while the modern world presents an environmental context drastically different to the ancestral one [2]. Thanks to vaccines and antibiotics, infant mortality is much reduced and life expectancy extended, and instead immune‐related disorders have become a major cause of morbidity and mortality, also for children [2]. A common misconception is that newborns are immunodeficient; however, they are immunologically competent, as evidenced by their ability to mount strong inflammatory responses to infections and effective memory responses to vaccines administered even on the first day of life (Hepatitis B, BCG, etc.) [3]. Newborns benefit from the transfer of maternal antibodies offering passive immunity [4] and the specificity of such maternal antibodies is an important determinant of infectious disease susceptibility as shown recently for neonatal sepsis caused by E. coli [5]. New immunological memory is lacking early in life as prior exposures are required, but in the first months of life the frequency of memory B and T cells increase steadily [6]. Recent observations show that innate immune responses mature rapidly to adult‐like levels within the first 3 months after birth (Arzoomand et al., unpublished observation).

### 1.1 Infectious Disease Susceptibility

Protection from invasive infections early in life is dependent on passive mechanisms such as barrier tissue integrity separating microbes from the inner tissues and active innate and adaptive defences localized to barrier surfaces. Also, the titers and specificity of maternal antibodies are important. The structural forms of protection evolve and mature but are underdeveloped in children born very preterm [7]. This, in combination with invasive procedures such as intravenous lines, invasive ventilation, and environmental exposures in the intensive care unit setting, is likely sufficient to explain the increased risks of invasive infections seen in babies born very preterm [8]. Also, in adult patients admitted to intensive care as compared to normal hospital wards, the likelihood of hospital‐acquired infections is higher [9].

Innate immune cell responses mature with cumulative exposures after birth, and specific factors such as vaccination with mycobacteria in the form of Bacille Calmette Guérin (BCG) vaccines have been proposed to accelerate this maturation process through epigenetic and immunometabolic adaptation, that is, trained immunity [10]. This can result in heterologous protection from various infectious diseases, beyond mycobacteria, in children born in areas where infectious disease burden is high [11]. The effects of trained immunity induced by BCG is less clear in industrialized societies where risks of infectious diseases are generally lower. A mild protective effect of early BCG on the development of immune mediated disease, including atopic dermatitis has been reported among high‐risk infants in Australia [12]. Thus, optimizing the development of immune‐microbe interactions and maturation of immune cells early in life requires deeper knowledge of what is optimal in each environmental context. This will require further studies across diverse populations.

### 1.2 Establishing Friendships for Life

The establishment of healthy immune‐microbe interactions is essential for fostering long‐term symbiotic relationships between the human host and the diverse microbiota colonizing the skin, lungs, and gastrointestinal tract. These microbes serve critical functions, including the metabolism of ingested food, the prevention of pathogen invasion through competitive inhibition, and the balanced “training” of the developing immune system to ensure reactivity without causing inflammatory illness [2]. The specific exposures driving innate immune cell maturation and the timing of microbial sensing in human newborns remain elusive. In mice, a weaning reaction has been described during which transient sensing of colonizing microbes occurs [13]. Weaning in humans is more gradual and is associated with diversification of the microbiota, reaching an adult‐like microbial composition in stool by 2 years of life [14]. Early cessation of breastfeeding (< 3 months) seems to impact this process of adaptation to microbial diversification negatively and is associated with increased risks of immune mediated disease later in life [15]. The optimal speed of development in industrialized societies as compared to more traditional environments, where risks of infection are likely higher, is unknown and an important focus of ongoing studies.

The first 3 months of life represent a critical window for immune development and establishment of immune‐microbe interactions [6, 16]. For example, antibiotic exposure during these initial months can have detrimental long‐term effects by perturbing the establishment of symbiosis, whereas similar exposure after 1 year of age does not carry the same risk [17]. Breastfeeding further supports this process by providing maternal nutrients optimized for beneficial microbes—giving them a competitive growth advantage over pathogens [18] —and delivering specific IgA antibodies that protect mucosal surfaces [19]. While paediatricians must remain sensitive to the fact that some mothers are unable to breastfeed for medical or social reasons, it is vital to promote these benefits and continue research into optimizing infant formulas and supplements to leverage the protective effects of these early immune‐microbe interactions.

### 1.3 Preterm Delivery

The transition from term to preterm delivery presents unique immunological challenges, as children born prematurely—sometimes as early as 22 weeks—face a significantly higher risk of complications and mortality. While the primary causes of death in these infants are infections and inflammatory conditions like necrotizing enterocolitis (NEC), the underlying reasons for this susceptibility are complex [20]. Although numerous studies have attempted to link infectious mortality to variations in immune cell numbers or functions when compared to term infants, they often struggle to distinguish between changes triggered by the initial cause of preterm delivery—such as chorioamnionitis or systemic inflammation—and those inherent to gestational age. The perceived “immunodeficiency” of preterm infants is also influenced by lower titers of maternal antibodies, which are primarily transferred across the placenta during the third trimester; however, a selective transfer of protective, neutralizing antibodies does occur earlier in pregnancy, providing even extremely preterm infants with some degree of coverage [4, 21]. Ultimately, the increased risk of infection in preterm babies may be better explained by non‐immunological factors, including invasive intensive care practices like ventilation and the use of central lines, as well as the inherent immaturity of physical barriers as described above. Taken together, there is little evidence to support the notion that preterm infants lack the capacity to mobilize immune defences when needed; rather, their vulnerability results from the underlying conditions, often inflammatory, leading to preterm delivery, their unique environmental exposures associated with intensive care and their physiological immaturity.

### 1.4 Boys and Girls

Among young children, especially those born preterm, boys are more likely to die from infectious diseases and suffer long‐term consequences [22]. The mechanisms of this sex difference are unknown, but immunological differences are likely to play a role. In general, it is well known that males and females exhibit striking differences in their immune systems and this is associated with different disease manifestations and risks. Females are overrepresented in most autoimmune diseases yet have better outcomes from most infectious diseases. Vaccine responses tend to be stronger in females as well. We recently showed that specific immune functions are dynamically regulated by sex hormones in adults, likely as a way of optimizing sex‐specific traits such as reproduction, muscle growth, and strength [23]. Because of such trade‐offs, infectious disease risks and manifestations will differ as well. Sex hormone regulation of immunity is likely to be less prominent in young, prepubertal children although a transient rise in sex steroids occurs by 2–3 months of age. Sex chromosomes regulate immune cells differently in males and females throughout life and recent work has shown broad cis‐ and trans‐regulatory effects of both the Y chromosome and the inactivated X chromosome on autosomal genes in human immune cells [24]. These findings point out that further studies are needed to better unravel the regulatory differences of immune cells between young boys and girls and in relation to maturation throughout the childhood period.

### 1.5 When to Worry

I often get questions from colleagues about what children to worry about and when to suspect inborn errors of immunity or abnormal inflammatory responses to infections, vaccines or other immunological stimuli. Consensus statements have been formulated by expert organizations such as the European Society for Immunodeficiency, ESID: https://esid.org/10‐warning‐signs‐of‐pid‐general/. Such signs of worry are centred around more frequent than expected infections, or more severe than expected presentations of infections, but also more general signs of worry such as failures to thrive which can have many causes, where immunodeficiency is one.

All children become colonized by microbes early in life and this is a process which has been perfected over thousands of years of human evolution. Excessive responses to colonizing microbes therefore represent a first possible sign of dysregulated immune cell circuits. Most newborn children present with self‐limiting inflammatory responses in the skin upon microbial colonization. Erythema toxicum neonatorum is a common example, limited by endogenous regulatory mechanisms such as the IL‐1 binding protein IL1RA. In infants lacking this protein (Deficiency of IL1RA, DIRA, OMIM: 147679), excessive inflammatory skin lesions are often the first sign and should warrant further investigation [25]. Similarly, upon colonization of the intestine, a mild inflammatory reaction is common, but excessive responses causing haematochezia and poor weight gain due to insufficient nutrient absorption should be further investigated for possible immunodeficiency or immune dysregulation such as IPEX disease [26]. Severe adverse reactions to common vaccines in childhood are another reason for concern that should merit consultation with experts in paediatric immunology [27]. Unexpectedly severe presentation following well known infections is a hallmark presentation of inborn errors of immunity as are opportunistic infections from microbes not causing significant infections in healthy children. The challenge with identifying such cases is that many inborn errors of immunity cause very narrow susceptibilities and children may go unnoticed by not encountering the specific pathogens to which they are increasingly susceptible. All children that raise suspicion of unusual presentation, especially when there is familial aggregation of cases or known heritability, should be consulted with immunology experts before deciding whether to perform further workup or not. All in all, the immune system of young children is fully capable in most cases, evolving with cumulative environmental exposures and adapting to the needs of the growing child. Further investigation into variations among populations, in relation to variable environmental contexts will be required to better prevent immune mediated diseases and give all children an optimal start in life.

## Author Contributions

**Petter Brodin:** conceptualization, writing – review and editing.

## Conflicts of Interest

The author declares no conflicts of interest.

## Data Availability

Data sharing not applicable to this article as no datasets were generated or analysed during the current study.
